# Radiation-induced temporal lobe injury after intensity modulated radiotherapy in nasopharyngeal carcinoma patients: a dose-volume-outcome analysis

**DOI:** 10.1186/1471-2407-13-397

**Published:** 2013-08-27

**Authors:** Ying Sun, Guan-Qun Zhou, Zhen-Yu Qi, Li Zhang, Shao-Min Huang, Li-Zhi Liu, Li Li, Ai-Hua Lin, Jun Ma

**Affiliations:** 1 State Key Laboratory of Oncology in Southern China, Department of Radiation Oncology, Cancer Center, Sun Yat-sen University, Guangzhou 510060, People’s Republic of China; 2 State Key Laboratory of Oncology in Southern China, Imaging Diagnosis and Interventional Center, Cancer Center, Sun Yat-sen University, Guangzhou 510060, People’s Republic of China; 3 Department of Medical Statistics and Epidemiology, School of Public Health, Sun Yat-sen University, Guangzhou 510060, People’s Republic of China

**Keywords:** Nasopharyngeal carcinoma, Temporal lobe injury, Intensity modulated radiotherapy, Radiation volume effect, Dose tolerance

## Abstract

**Background:**

To identify the radiation volume effect and significant dosimetric parameters for temporal lobe injury (TLI) and determine the radiation dose tolerance of the temporal lobe (TL) in nasopharyngeal carcinoma (NPC) patients treated with intensity modulated radiation therapy (IMRT).

**Methods:**

Twenty NPC patients with magnetic resonance imaging (MRI)-diagnosed unilateral TLI were reviewed. Dose-volume data was retrospectively analyzed.

**Results:**

Paired samples *t*-tests showed all dosimetric parameters significantly correlated with TLI, except the TL volume (TLV) and V_75_ (the TLV that received ≥75 Gy, *P* = 0.73 and 0.22, respectively). Receiver operating characteristic (ROC) curves showed V_10_ and V_20_ (*P* = 0.552 and 0.11, respectively) were the only non-significant predictors from V_10_ to V_70_ for TLI. D_0.5cc_ (dose to 0.5 ml of the TLV) was an independent predictor for TLI (*P* < 0.001) in multivariate analysis; the area under the ROC curve for D_0.5cc_ was 0.843 (*P* < 0.001), and the cutoff point 69 Gy was deemed as the radiation dose limit. The distribution of high dose ‘hot spot’ regions and the location of TLI were consistent.

**Conclusions:**

A D_0.5cc_ of 69 Gy may be the dose tolerance of the TL. The risk of TLI was highly dependent on high dose ‘hot spots’ in the TL; physicians should be cautious of such ‘hot spots’ in the TL during IMRT treatment plan optimization, review and approval.

## Background

Nasopharyngeal carcinoma (NPC) is common among Asians, especially in Southern China where the age-standardized incidence is 20–50 per 100,000 males [1]. Radical radiotherapy (RT) is the primary treatment modality for non-disseminated NPC due to its anatomic location and radiosensitivity; however, NPC radiotherapy is notoriously difficult due to the tumor’s invasive characteristics and proximity to critical structures. Late temporal lobe injury (TLI) due to radiotherapy is one of the most important dose-limiting factors and a frequently observed complication in NPC patients; TLI accounted for approximately 65% of deaths due to radiation-induced complications in patients who received conventional two-dimensional radiotherapy 2D-CRT, [2].

Intensity-modulated radiotherapy (IMRT) was a major break-through in the treatment of NPC, and it was capable of producing highly conformal dose distributions with steep dose gradients and complex isodose surfaces [3]. The design of appropriate dose constraints for the organs at risk (OAR) during the optimization of IMRT treatment plans can enable significantly better OAR sparing and reduce subsequent complications. However, the dose tolerances of many OARs, including the temporal lobe (TL), were poorly characterized. Furthermore, much of the existing data were based on the experience of clinicians in the 2D-CRT era, with a lack of solid clinical evidence [4]. There is a critical need for more accurate information about the tolerance of normal tissues to radiation in NPC patients receiving IMRT.

Therefore, the volumetric information for a cohort of NPC patients who developed unilateral TLI after treatment with radical IMRT was retrospectively reviewed, and dose–response relationships for the TL were investigated using a dose-volume-outcome analysis. We aimed to provide a practical guideline to improve the optimization of IMRT treatment plans, and determine the dose tolerance of the TL to achieve the greatest possibility of uncomplicated tumor control.

## Methods

### Patient selection

From January 2003 to December 2006, 506 newly diagnosed, non-distant-metastatic and histologically proven NPC patients were treated with IMRT. Twenty patients who completed a full course of IMRT whose follow-up magnetic resonance imaging (MRI, for at least 6 months post-radiotherapy) indicated unilateral TLI were included. Approval for retrospective analysis of the patient data was obtained from the ethics committee of Sun Yat-sen University Cancer Center. Informed consent was obtained from each patient.

All patients completed a pre-treatment evaluation including complete patient history, physical examination, hematology and biochemistry profiles, neck and nasopharynx MRI, chest radiography, abdominal sonography, and whole body bone scan using single photon emission computed tomography (SPECT). Positron emission tomography (PET)/CT was performed on 4/20 patients (20.0%). All patients were restaged according to the 2009 7th UICC/AJCC staging system [5].

### Radiotherapy techniques

Patients were immobilized in the supine position with a thermoplastic head and shoulder mask. Treatment planning CT was performed after administration of intravenous contrast medium, obtaining 3 mm slices from the head to the level 2 cm below the sternoclavicular joint. Target volumes were delineated using our institutional treatment protocol [6], in accordance with the International Commission on Radiation Units and Measurements reports 50 and 62 [7,8]. MRI was used to help define the parapharyngeal and superior extent of the tumor.

The contoured images were transferred to an inverse IMRT planning system (Corvus version 5.2; NOMOS Corp., Sewickley, PA, USA). The prescribed dose, as per the institutional protocol, was defined as: 68 Gy/30 fractions/6 weeks to the planning target volume (PTV) of the primary gross tumor volume (GTV-P), 60 to 64 Gy to the PTV of the nodal gross tumor volume (GTV-N), 60 Gy to the PTV of CTV-1 (i.e., high-risk regions), and 54 Gy to the PTV of CTV-2 (i.e., low-risk regions) and CTV-N (i.e., neck nodal regions). The nasopharynx and upper neck tumor volumes were treated by IMRT for the entire treatment course using a dynamic, multileaf, intensity-modulating collimator MIMiC (NOMOS Corp.). According to the complexity and length of the individual treatment target volume, five to seven 270° (from 225° to 135°, IEC conventions) arcs were used to treat the nasopharynx and upper neck. The treatment couch was moved between arcs at 2 cm intervals craniocaudally.

### MRI protocol

MRI was performed using a 1.5-Tesla system (Signa CV/i; General Electric Healthcare, Chalfont St. Giles, United Kingdom) examining the area from the suprasellar cistern to the inferior margin of the sternal end of the clavicle using a head-and-neck combined coil. T1-weighted fast spin-echo images in the axial, coronal and sagittal planes (repetition time, 500–600 ms; echo time, 10–20 ms), and T2-weighted fast spin-echo MRI in the axial plane (repetition time, 4,000-6,000 ms; echo time, 95–110 ms) were obtained before injection of contrast material. After intravenous injection of gadopentetate dimeglumine (0.1 mmol/kg body weight Gd-DTPA, Magnevist; Bayer-Schering, Berlin, Germany), spin-echo T1-weighted axial and sagittal sequences and spin-echo T1-weighted fat-suppressed coronal sequences were performed sequentially, using similar parameters to before injection. The section thickness was 5 mm with a 1 mm interslice gap for the axial plane, and 6 mm with a 1 mm interslice gap for the coronal and sagittal planes.

### Image assessment and diagnostic criteria for TLI

The MRI images were independently reviewed by two radiologistsand a clinician specializing in head-and-neck cancer; disagreements were resolved by consensus. MRI-detected TLI met one of the following criteria: a) white matter lesions, defined as areas of finger-like lesions of increased signal intensity on T2-weighted images; b) contrast-enhanced lesions, defined as lesions with or without necrosis on post-contrast T1-weighted images with heterogeneous signal abnormalities on T2-weighted images; c) cysts, round or oval well-defined lesions of very high signal intensity on T2-weighted images with a thin or imperceptible wall [9].

### TL re-delineation and data collection

The TL volume delineated in the treatment plan failed to cover the regions overlapping the target volume, due to an inherent limitation of the Corvus system. CERR DICOM-RT toolbox (version 3.0 beta 3; School of Medicine, Washington University, St. Louis, USA) was used to re-delineate the TL and collect the following dosimetric parameters: mean dose, volume of the TL (TLV), D_0.1CC_ (the dose to 0.1 ml of the TL volume), D_0.5CC_, D_1CC_, D_5CC_, D_10CC_, D_15CC_, D_20CC_, D_25CC_, D_30CC_, D_35CC_, D_40CC_, D_1_ (the dose to 1% of the TL volume), D_5_, D_10_, D_33_, D_35_, D_40_, D_45_, D_50_, D_55_, D_60_, V_10_ (the volume of the TL that received more than 10 Gy), V_20_, V_25_, V_30_, V_35_, V_40_, V_45_, V_50_, V_55_, V_60_, V_65_, V_70_, V_75_.

### Follow up and statistical analysis

Patients were followed up at least every three months in the first three years and every six months thereafter. The median follow-up of this cohort of patients was 65.5 months (range 30.1 to 97.1 months), and the final follow-up MRI was performed on May 18th, 2012. Routine follow-up care included a complete head and neck examination, hematology and biochemistry profiles, chest radiography and abdominal sonography. Follow-up MRI of the neck and/or nasopharynx was performed for cases with suspected tumor recurrence or radiotherapy-induced complications.

All analyses were performed using SPSS software version 13.0 (SPSS, Chicago, IL, USA). Dosimetric parameters in the paired contralateral TLs were compared using paired samples *t*-tests. Cutoff points for significant dosimetric parameters in the receiver operating characteristic (ROC) analysis were used to create the TL dose-volume histogram (DVH). Significant dosimetric parameters in the paired samples *t*-test were further tested in multivariate analyses using the Cox proportional hazards model. Independent significant factors were assessed using ROC curves to estimate the TL dose tolerance. Two-sided *P* values ≤0.05 were considered statistically significant.

## Results

### Clinical characteristics of the NPC patients

Twenty patients who developed unilateral TLI were included in this study. The male/female ratio was 4:1 (16 males, 4 females); median age was 42.5 years (range, 25–55 years). All patients had World Health Organization (WHO) type II or III disease; 18 patients with T3/T4 disease received chemotherapy, and two with T1/T2 disease received IMRT only. The patients developed TLI within a median latency of 33.6 months (range, 25.1 to 56.9 months) from commencement of primary radiotherapy. Histological confirmation of radiation necrosis was available in one patient who underwent temporal lobectomy. The characteristics of the 20 NPC patients are presented in Table 1.

**Table 1:** Characteristics of the 20 NPC patients who developed unilateral temporal lobe injury

**Case**	**Gender**	**Age (years)**	T stage*	Stage*	**OTT (days)**	**CT**	**TLI latency (months)**
1	male	54	3	III	38	yes	40.27
2	male	40	4	IVa	41	yes	25.77
3	male	54	4	IVa	41	yes	31.47
4	male	42	4	IVa	39	yes	35.37
5	male	37	4	IVa	78	yes	38.13
6	male	55	3	III	47	yes	35.57
7	male	40	4	IVa	38	yes	25.13
8	male	39	4	IVa	46	yes	25.40
9	male	43	4	IVa	46	yes	27.27
10	male	36	3	III	48	yes	37.90
11	female	25	4	IVa	40	yes	26.40
12	male	50	4	IVa	42	yes	31.90
13	male	44	4	IVa	44	yes	25.13
14	male	43	4	IVa	38	yes	27.17
15	male	41	4	IVa	39	yes	48.43
16	male	52	2	II	42	no	27.67
17	female	34	4	IVa	45	yes	46.33
18	female	33	4	IVa	49	yes	44.70
19	male	52	1	I	40	no	50.70
20	female	51	4	IVa	43	yes	56.87

*Abbreviations*: *OTT* Overall treatment time, *CT* Chemotherapy.

*According to the American Joint Committee on Cancer, 7th edition.

### Significant dosimetric parameters and dose-volume histogram

The 36 dosimetric parameters (see materials and methods) were compared in each affected TL and the corresponding unaffected TL. Paired samples *t*-tests showed all parameters, except for TLV and V_75_ (*P* = 0.73 and 0.22 respectively), were significantly associated with TLI (Table 2).

**Table 2:** Comparison of dosimetric parameters in the contralateral TLs for 20 NPC patients with unilateral TLI

**Variable**	**Mean difference**	**SE**	**95% CI**		***t***	***P***-**Value**
			**Upper**	**Lower**		
TLV	−0.48	1.36	−3.33	2.37	−0.36	0.73
D _mean_	4.53	0.78	2.90	6.15	5.82	0.00
D_0.1CC_*	10.18	1.60	6.84	13.52	6.38	0.00
D_0.5CC_	11.19	1.76	7.50	14.88	6.35	0.00
D_1CC_	11.88	1.92	7.88	15.89	6.21	0.00
D_5CC_	13.95	2.02	9.71	18.20	6.88	0.00
D_10CC_	14.23	2.19	9.64	18.81	6.49	0.00
D_15CC_	13.68	2.35	8.77	18.59	5.83	0.00
D_20CC_	10.73	2.07	6.39	15.07	5.18	0.00
D_25CC_	8.26	1.83	4.42	12.09	4.50	0.00
D_30CC_	6.45	1.52	3.26	9.63	4.24	0.00
D_35CC_	4.56	1.18	2.08	7.03	3.86	0.00
D_40CC_	3.37	0.89	1.50	5.24	3.77	0.00
D_1_^§^	12.08	1.88	8.14	16.02	6.42	0.00
D_5_	14.27	2.01	10.05	18.50	7.07	0.00
D_10_	14.13	2.12	9.70	18.56	6.68	0.00
D_15_	12.31	2.13	7.84	16.78	5.77	0.00
D_20_	9.64	1.91	5.63	13.65	5.03	0.00
D_25_	7.07	1.43	4.08	10.07	4.94	0.00
D_30_	4.79	1.01	2.68	6.91	4.74	0.00
D_33_	3.91	0.87	2.09	5.74	4.49	0.00
D_35_	3.46	0.81	1.77	5.15	4.28	0.00
D_40_	2.53	0.64	1.18	3.88	3.93	0.00
D_45_	1.85	0.51	0.78	2.91	3.62	0.00
D_50_	1.49	0.41	0.64	2.34	3.67	0.00
D_55_	1.20	0.35	0.46	1.94	3.38	0.00
D_60_	1.04	0.35	0.31	1.76	2.99	0.01
V_10_^♀^	3.67	1.37	0.79	6.55	2.67	0.02
V_20_	4.87	0.91	2.96	6.78	5.34	0.00
V_25_	5.91	0.99	3.83	7.98	5.97	0.00
V_30_	7.06	1.11	4.73	9.38	6.35	0.00
V_35_	6.67	1.24	4.06	9.28	5.35	0.00
V_40_	8.11	1.31	5.37	10.84	6.21	0.00
V_45_	7.94	1.34	5.15	10.74	5.95	0.00
V_50_	7.82	1.33	5.03	10.60	5.88	0.00
V_55_	7.31	1.26	4.67	9.95	5.79	0.00
V_60_	6.23	1.15	3.82	8.64	5.40	0.00
V_65_	4.58	0.96	2.56	6.60	4.75	0.00
V_70_	2.59	0.71	1.10	4.09	3.63	0.00
V_75_	0.86	0.34	0.14	1.58	2.50	0.22

*Abbreviations*: *TLs* Temporal lobes, *TLI* Temporal lobe injury, *CI* Confidence interval, *TLV* Volume of an individual temporal lobe, *D*_*mean*_ Mean dose to temporal lobe, *D*_*max*_ Maximum dose to temporal lobe.

*D_0.1CC_ is the dose to 0.1 ml of the temporal lobe volume; other absolute volumes are indicated in a similar manner.

§D_1_ is the dose to 1% of the temporal lobe volume; Other percentage volumes are indicated in a similar manner.

♀V_10_ is the absolute volume of the temporal lobe that received more than 10 Gy; other doses are indicated in a similar manner.

**Figure 1:**
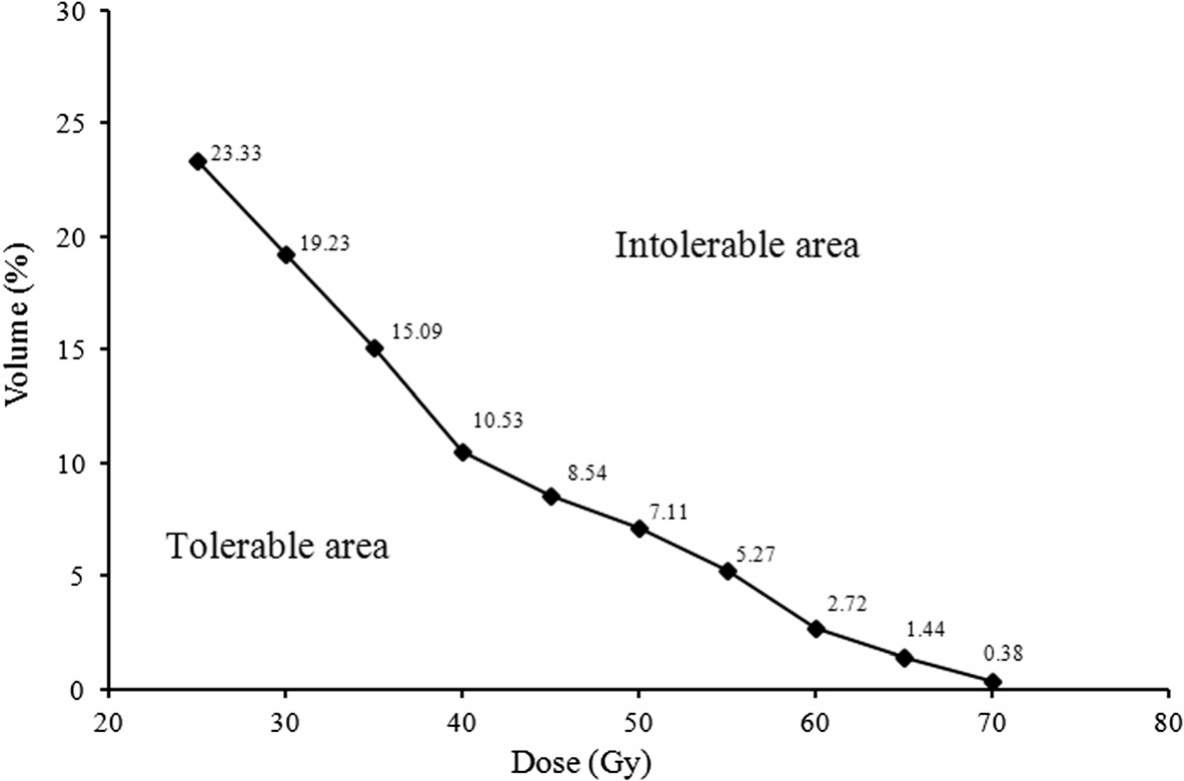
**Temporal lobe (TL) irradiation tolerance curve expressed as a cumulative dose-volume histogram.** The histogram was created using the cutoff points in Table 3. The area under the dose–volume histogram curve was assumed to be tolerable, and the area over the curve, intolerable. The sensitivity and specificity for prediction of TLI ranged from 0.70 to 0.85, and 0.80 to 0.85 (see Table 3).

For the significant dosimetric parameters (in paired-samples *t*-tests) from V_10_ to V_70_, ROC curves demonstrated that V_10_ and V_20_ were the only non-significant factors for TLI (area under the ROC curves, 0.555 and 0.647; *P* = 0.552 and 0.11, respectively; Table 3). The cutoff points for the dose tolerance of the TL for each significant parameter were selected using *P* < 0.05 and Youden’s index. The significant parameters and cutoff points are shown in Table 3 as V_25_ (23.325%), V_30_ (19.225%), V_35_ (15.09%), V_40_ (10.53%), V_45_ (8.537%), V_50_ (7.114%), V_55_ (5.27%), V_60_ (2.72%), V_65_ (1.44%), and V_70_ (0.379%). A cumulative DVH for the dose tolerance of TL was drawn using the significant cutoff points (Figure 1). The area under the ROC curve was designated tolerance, and the area above the curve, intolerance. The curve showed an increasing probability of TLI with increasing dose.

**Table 3:** **Summary of temporal lobe radiation tolerance expressed as V**_**10**-**75**_**using paired t**-**tests and ROC curve**

	**Area under ROC curve**	***β***	***P***	**Lower limit**	**Upper limit**	**Cutoff point (%)**	**Sensitivity**	**Specificity**
V_10_*	0.555	0.093	0.552	0.374	0.736			
V_20_	0.647	0.091	0.11	0.47	0.825			
V_25_	0.687	0.087	0.042	0.517	0.858	23.325	0.7	0.75
V_30_	0.74	0.084	0.009	0.575	0.905	19.225	0.75	0.8
V_35_	0.729	0.087	0.013	0.558	0.899	15.09	0.75	0.8
V_40_	0.78	0.08	0.002	0.623	0.937	10.53	0.8	0.8
V_45_	0.798	0.076	0.001	0.649	0.946	8.537	0.8	0.85
V_50_	0.825	0.7	0.000	0.687	0.936	7.114	0.8	0.9
V_55_	0.855	0.062	0.000	0.733	0.977	5.27	0.8	0.9
V_60_	0.85	0.066	0.000	0.72	0.98	2.72	0.85	0.85
V_65_	0.85	0.068	0.000	0.716	0.984	1.44	0.85	0.85
V_70_	0.835	0.071	0.000	0.696	0.974	0.379	0.85	0.85

*Abbreviation*: *ROC* Receiver operating characteristic.

*V_10_ is the volume of the temporal lobe that received more than 10 Gy; other volumes are indicated in a similar manner.

### Independent indicators and dose tolerance of the TL with respect to TLI

Multivariate analysis by forward elimination of insignificant explanatory variables was performed to adjust for various factors; all significant parameters from the paired samples *t*-tests were include as covariates. D_0.5cc_ was the only independent predictor of TLI in the Cox regression model (β = −0.17, SE = 0.05, RR [Relative Risk] = 0.84, 95% CI [Confidence Interval] for RR = [0.76, 0.93], *P* < 0.001).

**Figure 2:**
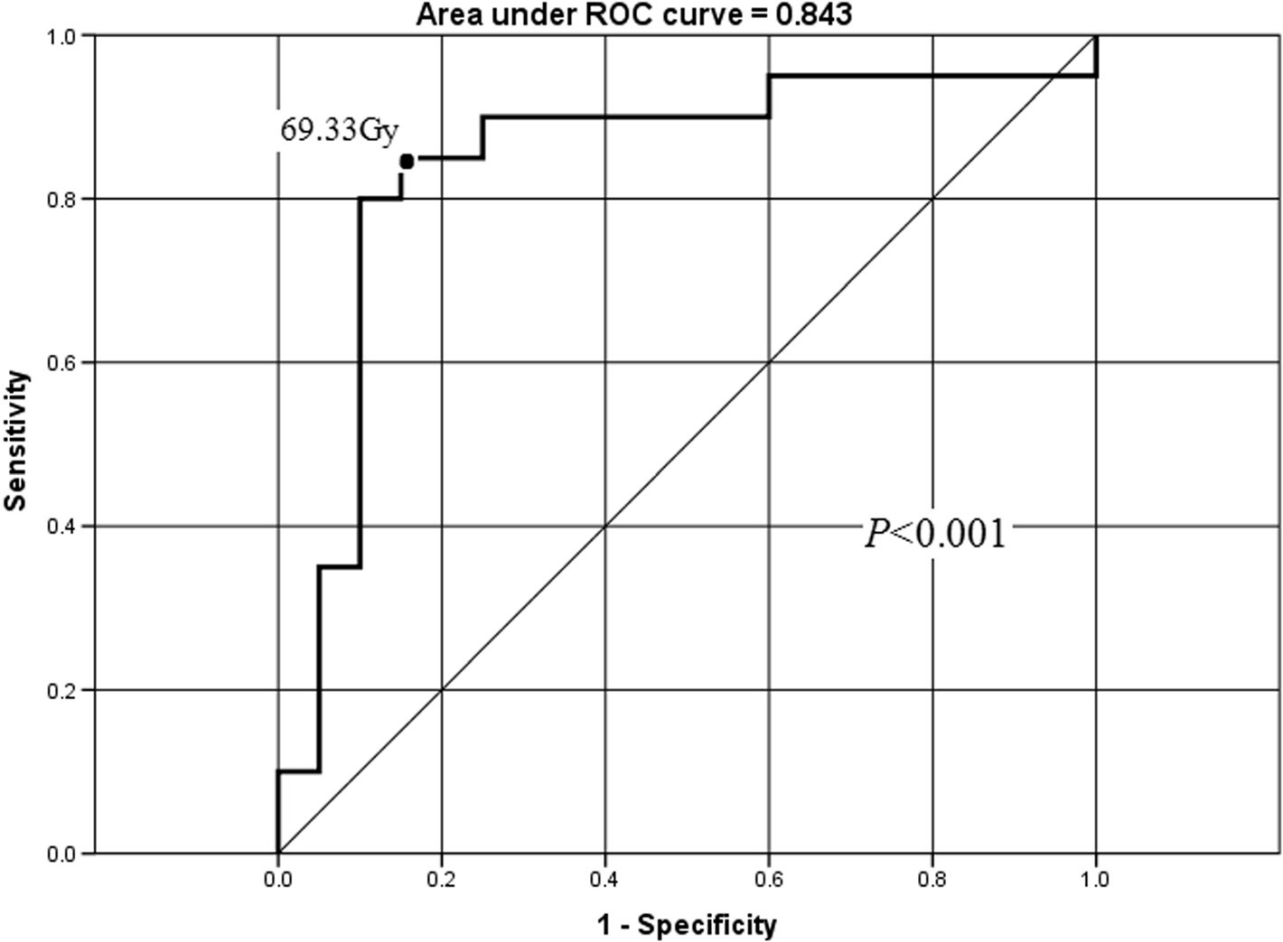
**Receiver operating characteristic (ROC) curve for D**_**0.5cc **_**(dose to 0.5 ml of temporal lobe volume).** The cutoff point for D_0.5cc_ (as the temporal lobe dose tolerance) was determined as 69 Gy for NPC patients treated with IMRT. At a D_0.5cc_ of 69 Gy, the sensitivity and specificity for the prediction of radiation-induced temporal lobe injuries (TLI) were 0.85 and 0.85, respectively.

We determined the dose tolerance of the TL using ROC curves, in terms of the independent significant variable D_0.5cc_. The area under the ROC curve was 0.843 for D_0.5cc_ (*P* < 0.001; Figure 2). From Figure 2, it would be appropriate to consider a D_0.5cc_ of 69 Gy as the dose tolerance of the TL (sensitivity, 0.85; specificity, 0.85). The mean D_0.5cc_ for affected TLs was 73.53 Gy ± 7.34 Gy, and 62.33 Gy ± 7.97 Gy for unaffected TLs.

### Coincidence of ‘hot spots’ with the location of TLI

**Figure 3:**
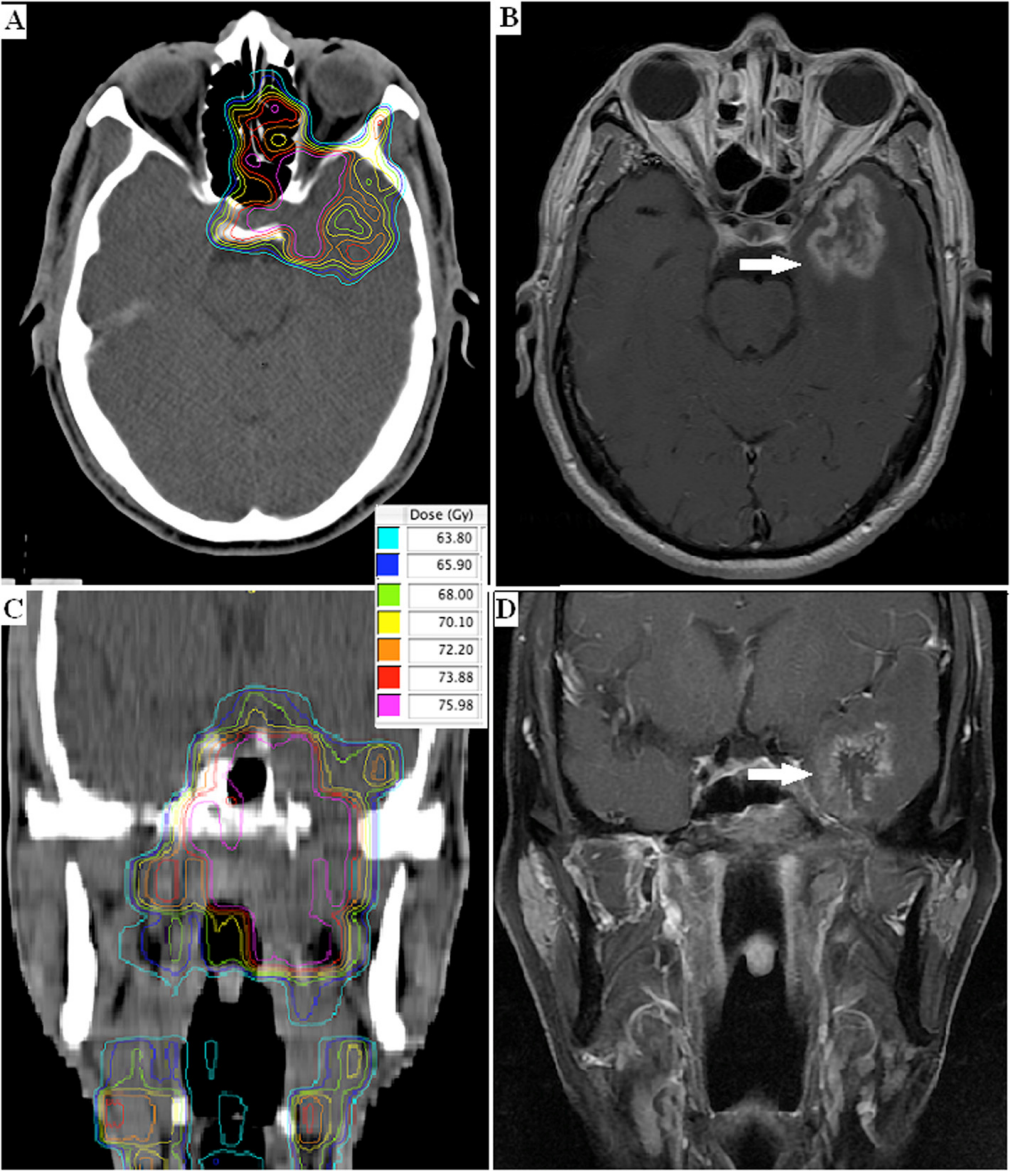
**Dose distribution and corresponding necrosis nidus within the temporal lobes (arrow).** Axial **(A** and **B)** and coronal **(C** and **D)** MRI images of a 62-yr-old NPC patient (patient 1 in Table 1).

Analysis of the relationship between high dose ‘hot spot’ regions in the TL and the location of TLI was performed. As shown in the transverse images (Figure 3A) from a representative patient (case 1 in Table 1), the volume receiving a dose over 69 Gy in the left TL was highly concordant with the location of necrosis nidus, which occurred at almost exactly the same site (Figure 3B). In a similar manner, the coronal images in Figure 3C and D demonstrate the consistency of this ‘hot spot’ and the location of TLI.

## Discussion

Radiation-induced TLI is usually devastating to patients; however, there is a poor understanding of TLI in NPC patients treated with IMRT. Knowledge of the dose tolerance of the TL is essential, in order to predict the safety of IMRT treatment plans. This retrospective study analyzed the dose–response relationships for the TL, with the purpose of improving the understanding of TLI and thus optimizing IMRT treatment planning for NPC patients.

### Volume effect in the TL

The volume effect in normal organs is a major concern in radiotherapy. Withers et al. originally introduced the concept of tissue radiation tolerance based on functional subunits (FSUs), which can be either arranged in parallel or in series. The risk of complications depends on the total dose distribution within the organ in parallel organs, and on individual high dose ‘hot spots’ in series organs [10].

With respect to radiation-induced side-effects in the brain, in 1991 investigators pooled their clinical experience, judgment and information regarding partial organ dose tolerances, and suggested the dose to one-third of the brain was the major limiting parameter [4]. Most other previous studies have also implied that the total dose to the total irradiated brain volume was the most important dosimetric factor for predicting the risk of TLI [11]. However, it was difficult to distinguish the influence of dosimetric parameters from complex host-related factors in these previous studies. Therefore, patients who experience unilateral TL damage provided a unique opportunity for studying dosimetric predictors.

In the current analysis, all DVH-based variables (except for TLV and V_75_) correlated with the development of TLI in univariate analysis. Given the possibility of confounding interactions, multivariate analysis was performed to determine significant, independent predictive factors. The D_0.5cc_ ‘hot spot’ was identified as the most valuable predictor, which implied that TLI occurred as a serial complication, and also that the risk of TLI was most significantly related to the ‘hottest’ portion of the DVH; the dose distribution within the entire organ may be less relevant.

The difference between our observations and previous studies may partially be explained by the use of different radiation techniques. Most previous studies were based on 2D-CRT, for which detailed dose-volume parameters were not available. When using 2D-CRT, the entire brain dose, which was easier to determine and indirectly related to the maximum irradiation dose, seemed to correlate with the occurrence of radiation-induced brain injury [4]. However, according to our data, the TL was better described as a serial organizational structure. Since different areas of the TL perform specific functions [12], the radiation volume effects may also depend on the precise areas irradiated.

### Dose tolerance of the TL

Dose tolerances of the brain were first specified by Emami et al. in 1991. For irradiation of one-third of the brain, the TD5 was estimated as 60 Gy [4]; however, this estimate appeared overly conservative in many later studies [13-15]. In 2010, the QUANTEC (Quantitative Analysis of Normal Tissue Effects in the Clinic) study reported that a 5% and 10% risk of symptomatic radiation necrosis was predicted to occur at a biological effective dose of 120 Gy (range, 100–140) and 150 Gy (range, 140–170), respectively (corresponding to 72 Gy [range, 60–84] and 90 Gy [range, 84–102] in 2 Gy fractions) [16]. Although the QUANTEC study did not specify the volume limits these constrains were based on, and the conclusions were drawn from heterogeneous data (i.e., different target volumes, endpoints, sample sizes and brain regions), its observations agreed with our result that the dose tolerance of the TL was 69 Gy when 0.5 ml of the volume was irradiated. Similarly, a recent retrospective analysis of 870 NPC patients revealed that IMRT with a D_max_ < 68 Gy or D_1cc_ < 58 Gy for the TL was relatively safe [17].

The radiation damage occurring after carbon ion therapy appeared to be similar to that of proton therapies. Schlampp et al. calculated the relative biological effectiveness of carbon ion therapy in 118 temporal lobes in 59 patients, and reported that the D_max_ (V_1cc_) was predictive for radiation-induced TLI. They estimated the TD5 and TD50 dose tolerance of the brain as D_max_ values of 68.3 ± 3.3 Gy and 87.3 ± 2.8 Gy, respectively [18].

However, although the term tolerance is used frequently when discussing radiotherapy toxicity, it is important to realize that there is no dose below which the complication rate is zero: in other words, there is no clear-cut dose tolerance limit. In addition, radiation tolerance may vary depending on patient- and tumor-specific characteristics, as well as treatment modifications.

### High dose regions in the TL

In clinical practice, protection of the OARs including the spinal cord, brainstem, optic nerves and chiasm is deemed critical in NPC, and expanded OAR margins, termed planning organ at risk volumes (PRVs), are usually created to ensure these OARs do not receive excessive irradiation. As a result, late radiotherapy-induced effects have been successfully minimized or reduced for these OARs [19,20]. For example, in a study from Hong Kong, none of the 422 NPC patients developed damage to the optic nerve, optic chiasm, brain stem or spinal cord [19].

However, most radiotherapy centers, including our own institution, have not yet established an OAR dose limit for the TL. One could postulate that the relatively high dose delivered to the TL, compared to other critical normal tissues, could be due to the lack of an established TL dose limit. As NPC is located in the midline, with dose constraints superiorly for the optic nerve and optic chiasm, and constraints posteriorly for the brainstem, the use of fields from predominantly superior or posterior directions are limited in clinical practice. Hence lateral approaches are weighted higher to accomplish high-dose target coverage while complying with the OAR-defined dose limitations.

## Conclusions

We performed a retrospective dose-volume-outcome analysis for the TL in NPC patients treated with IMRT. The data indicates that radiation-induced TLI is a serial complication, with the ‘hottest’ dose in the TL the most important factor. We suggest a D_0.5CC_ limit of 69 Gy for the TL. This study provides valuable insight into the risk factors for TLI, and will help to optimize NPC treatment planning to improve tumor control and avoid side effects.

## Abbreviations

TLI: Temporal lobe injury; TL: Temporal lobe; NPC: Nasopharyngeal carcinoma; IMRT: Intensity modulated radiation therapy; TLV: TL volume; ROC: Receiver operating characteristic; RT: Radiotherapy; TLI: Late temporal lobe injury; 2D-CRT: Conventional two-dimensional radiotherapy; OARs: Organs at risk; MRI: Magnetic resonance imaging; SPECT: Single photon emission computed tomography; PET: Positron emission tomography; PTV: Planning target volume; GTV-P: Primary gross tumor volume; GTV-N: Nodal gross tumor volume; DVH: Dose-volume histogram; WHO: World Health Organization; CI: Confidence interval; PRVs: Planning organ at risk volumes.

## Competing interests

The authors declare that they have no competing interests.

## Authors’ contributions

The authors contributions are the following: YS and GQZ contributed with literature research, study design, data collection, data analysis, interpretation of findings and writing of the manuscript. ZYQ, LZ, SMH contributed with data collection. LZL and LL contributed with reviewing MR images. AHL contributed with data analyses. JM contributed with data collection, study design, critical review of data analyses, interpretation of findings and critical edit of the manuscript. All authors read and approved the final manuscript.

## Pre-publication history

The pre-publication history for this paper can be accessed here:

http://www.biomedcentral.com/1471-2407/13/397/prepub
